# Small cell transformation of *ROS1* fusion-positive lung cancer resistant to ROS1 inhibition

**DOI:** 10.1038/s41698-020-0127-9

**Published:** 2020-08-03

**Authors:** Jessica J. Lin, Adam Langenbucher, Pranav Gupta, Satoshi Yoda, Isobel J. Fetter, Marguerite Rooney, Andrew Do, Marina Kem, Kylie Prutisto Chang, Audris Y. Oh, Emily Chin, Dejan Juric, Ryan B. Corcoran, Ibiayi Dagogo-Jack, Justin F. Gainor, James R. Stone, Jochen K. Lennerz, Michael S. Lawrence, Aaron N. Hata, Mari Mino-Kenudson, Alice T. Shaw

**Affiliations:** 1 Department of Medicine, Massachusetts General Hospital, Boston, MA USA; 2 Harvard Medical School, Boston, MA USA; 3 Department of Pathology, Massachusetts General Hospital, Boston, MA USA

**Keywords:** Lung cancer, Lung cancer

## Abstract

Histologic transformation from non-small cell to small cell lung cancer has been reported as a resistance mechanism to targeted therapy in *EGFR*-mutant and *ALK* fusion-positive lung cancers. Whether small cell transformation occurs in other oncogene-driven lung cancers remains unknown. Here we analyzed the genomic landscape of two pre-mortem and 11 post-mortem metastatic tumors collected from an advanced, *ROS1* fusion-positive lung cancer patient, who had received sequential ROS1 inhibitors. Evidence of small cell transformation was observed in all metastatic sites at autopsy, with inactivation of *RB1* and *TP53*, and loss of *ROS1* fusion expression. Whole-exome sequencing revealed minimal mutational and copy number heterogeneity, suggestive of “hard” clonal sweep. Patient-derived models generated from autopsy retained features consistent with small cell lung cancer and demonstrated resistance to ROS1 inhibitors. This case supports small cell transformation as a recurring resistance mechanism, and underscores the importance of elucidating its biology to expand therapeutic opportunities.

## Introduction

Gene fusions involving the ROS1 proto-oncogene 1 (*ROS1*) are oncogenic drivers across multiple tumor types, including non-small cell lung cancer (NSCLC)^1–4^. Targeted therapy with ROS1 tyrosine kinase inhibitors (TKIs) such as crizotinib and entrectinib usually yields deep and durable tumor responses in *ROS1* fusion-positive (*ROS1*+) lung cancer^5–9^. Despite initial efficacy, however, most tumors eventually become refractory to ROS1 inhibition. Initial studies of crizotinib resistance in *ROS1*+ NSCLC have focused on secondary ROS1 kinase domain mutations (KDMs), such as ROS1 G2032R, which confers high-level resistance to the majority of clinically available ROS1 TKIs^10–13^. Yet, approximately two-thirds of TKI-resistant *ROS1*+ lung cancers do not harbor ROS1 KDMs, and are instead driven by ROS1-independent mechanisms of resistance^11–14^. Insights into ROS1-independent resistance mechanisms remain limited.

Tumor lineage changes, including epithelial-to-mesenchymal transition (EMT) or histologic transformation into small cell lung cancer (SCLC), represent a target-independent TKI resistance mechanism^15^. Small cell transformation has been identified in ~3–10% of TKI-resistant, *EGFR*-mutant NSCLC, and is associated with an aggressive clinical phenotype, limited therapy options, and poor prognosis^16–21^. There are also a few case reports of small cell transformation in TKI-resistant, *ALK* fusion-positive lung cancer^22–29^. Whether small cell transformation can mediate TKI resistance in other molecular subsets of NSCLC is unknown.

Here we examine multiple serial and metastatic tumor samples collected pre- and post mortem from a patient with advanced *ROS1*+ lung cancer who had received multiple ROS1 TKIs. We show that resistance was due to small cell transformation and describe the genomic landscape and clonal evolution of the transformed lung cancer.

## Results

### Clinical case

**Fig. 1:**
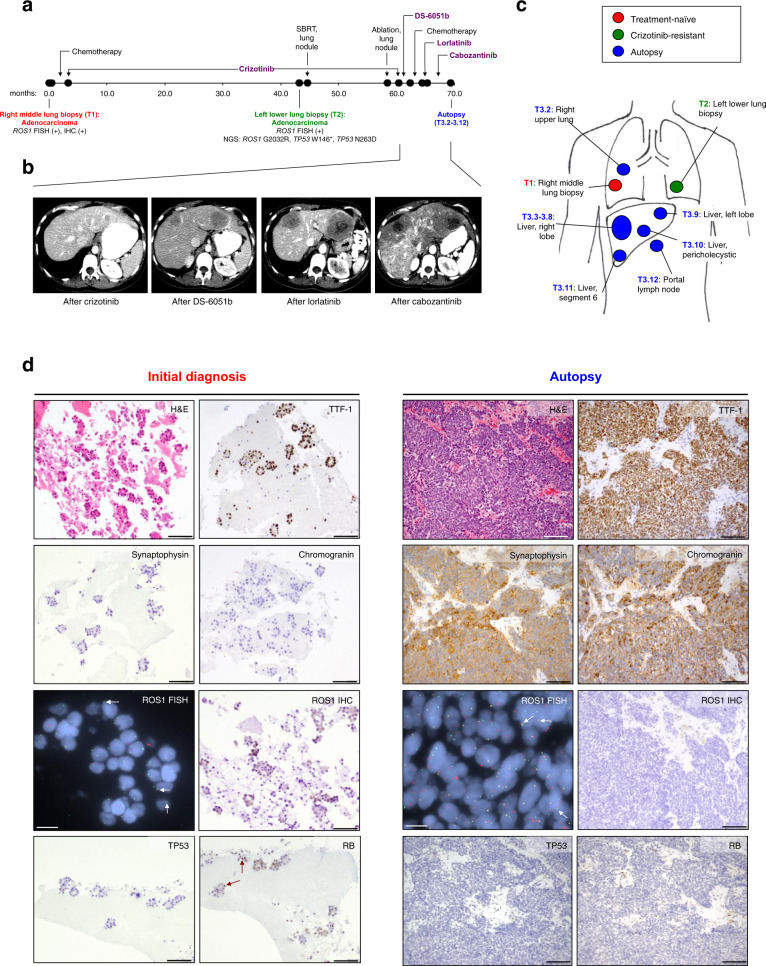
Clinical history and histopathologic findings of small cell transformation. **a** Timeline demonstrating the treatment course of the patient and time points of tumor (T) collection. Numbers represent months since initial diagnosis. SBRT stereotactic body radiation therapy, FISH fluorescence in situ hybridization, IHC immunohistochemistry, NGS next-generation sequencing. **b** Representative axial computed tomography images demonstrating the progression of hepatic metastases during the disease course. **c** Schematic of the tumor samples collected. **d** Immunostains of the treatment-naive lung primary demonstrating thyroid transcription factor-1 (TTF-1)-positive adenocarcinoma, and of the representative autopsy tumor specimen demonstrating small cell morphology with positive stains for synaptophysin and chromogranin. H&E hematoxylin and eosin. *ROS1* FISH demonstrates that the *ROS1* fusion is retained in both treatment-naive and autopsy samples; representative split signals indicative of *ROS1* fusion are highlighted by white arrows. ROS1 IHC illustrates detectable ROS1 protein expression in the treatment-naive tumor, but undetectable ROS1 expression in the small cell tumor. Immunostains for TP53 and RB1 reveal wild-type RB1 (red arrows indicating positive nuclear staining) and loss of TP53 expression in the treatment-naive tumor, and lack of RB1 and TP53 expression in the small cell-transformed tumor. The scale bar represents 100 µm in all panels except for the *ROS1* FISH panel, in which it represents 30 µm. Magnification: ×200 for H&E and TTF-1, synaptophysin, chromogranin, and ROS1, and ×400 for TP53 and RB, of the initial biopsy sample; ×100 for H&E and TTF-1, synaptophysin, chromogranin, ROS1, TP53, and RB immunostains of the autopsy sample.

A 32-year-old woman of South Asian descent presented with persistent dry cough and decreased exercise tolerance (Fig. 1a). Computed tomography (CT) scan of the chest revealed extensive multifocal areas of mass-like consolidation and nodules bilaterally. CT of the abdomen and pelvis and brain magnetic resonance imaging did not show evidence of distant metastases. Core biopsy of the right middle lobe lung tumor (T1, Fig. 1a) demonstrated adenocarcinoma with mixed lepidic and micropapillary patterns. By immunostaining, tumor cells were positive for cytokeratin 7 (CK7) and thyroid transcription factor-1 (TTF-1).

The patient received four cycles of chemotherapy (carboplatin, pemetrexed, and bevacizumab), and showed clinical and radiologic improvement. Molecular testing of the lung tumor revealed a *ROS1* rearrangement by fluorescence in situ hybridization (FISH). In light of this result, she enrolled in a phase I trial of crizotinib, and received crizotinib 250 mg twice a day with tumor reduction (best response of 30.1% reduction according to RECIST version 1.1). After 43 months, imaging revealed isolated progression of a left lower lobe lung nodule, with continued response elsewhere. Repeat biopsy of this lung nodule (T2, Fig. 1a) confirmed adenocarcinoma. Targeted next-generation sequencing (NGS) of 39 cancer-associated genes [SNaPshot NGS version 1; Massachusetts General Hospital (MGH), Boston, MA] detected the presence of a G2032R (c.6094G > A) mutation in the ROS1 kinase domain^10^. Additionally, two single-nucleotide variants were detected in *TP53*: W146* (c.437G > A) and N263D (c.787A > G). The latter, *TP53* N263D variant (rs72661119), has been reported in 10 of 13,894 individuals in the South Asian population and is likely a polymorphism^30^. The patient was treated with stereotactic body radiation therapy (SBRT) to the growing lung nodule and continued on crizotinib. Subsequent scans showed growth of an adjacent left lower lobe lung nodule, which was not biopsied, but treated with microwave ablation (Fig. 1a).

Repeat imaging after 57 months on crizotinib demonstrated two new hepatic metastases (Fig. 1b). Crizotinib was discontinued. The patient was treated with DS-6051b, an investigational ROS1/tropomyosin receptor kinase TKI^31^, and had primary progression with new, enlarging hepatic metastases. She subsequently received short courses of chemotherapy (carboplatin, pemetrexed, bevacizumab), lorlatinib (a ROS1/ALK TKI)^8^, and cabozantinib (multitargeted TKI with ROS1 activity)^32,33^, none of which induced a tumor response (Fig. 1b). Ultimately, treatment was discontinued and the patient died 69 months after her initial diagnosis.

### Histopathologic characterization of transformed SCLC

An autopsy was performed per the request of the patient’s family. Metastatic tumor samples were collected from the lung, liver, and portal lymph node (T3.2–3.12, Fig. 1c). Of note, six regions were sampled from a dominant right hepatic lobe metastasis (T3.3–3.8, Fig. 1c); one representative region was sampled from each of the remaining metastatic sites.

Histopathology review of all of the autopsy samples revealed tumor cells with scant cytoplasm, finely dispersed chromatin and inconspicuous nucleoli, morphologically distinct from the patient’s prior treatment-naive and crizotinib-resistant lung biopsy specimens (Fig. 1d). By immunohistochemical staining, the tumor cells were positive for CK7, TTF-1, and chromogranin and synaptophysin, consistent with the diagnosis of small cell lung carcinoma.

*ROS1* FISH confirmed the presence of a *ROS1* rearrangement in all autopsy tumor samples (Fig. 1d). However, by immunohistochemistry (IHC), all autopsy samples lacked ROS1 protein expression (Fig. 1d). Further, targeted RNA-based NGS analysis evaluating for fusion transcripts involving 12 cancer-related genes (Solid Fusion Assay, MGH, Boston, MA) did not detect a *ROS1* fusion in two autopsy samples that were analyzed (T3.2, T3.12), indicating loss of ROS1 expression at the RNA level. In contrast, an *SLC34A2-ROS1* fusion transcript and ROS1 protein expression by IHC were detected in the treatment-naive tumor (T1). Therefore, despite the continued presence of *ROS1* rearrangement at the DNA level, transformation to SCLC resulted in loss of *ROS1* fusion expression.

### Genomic landscape of transformed, *ROS1* fusion-positive SCLC

**Fig. 2:**
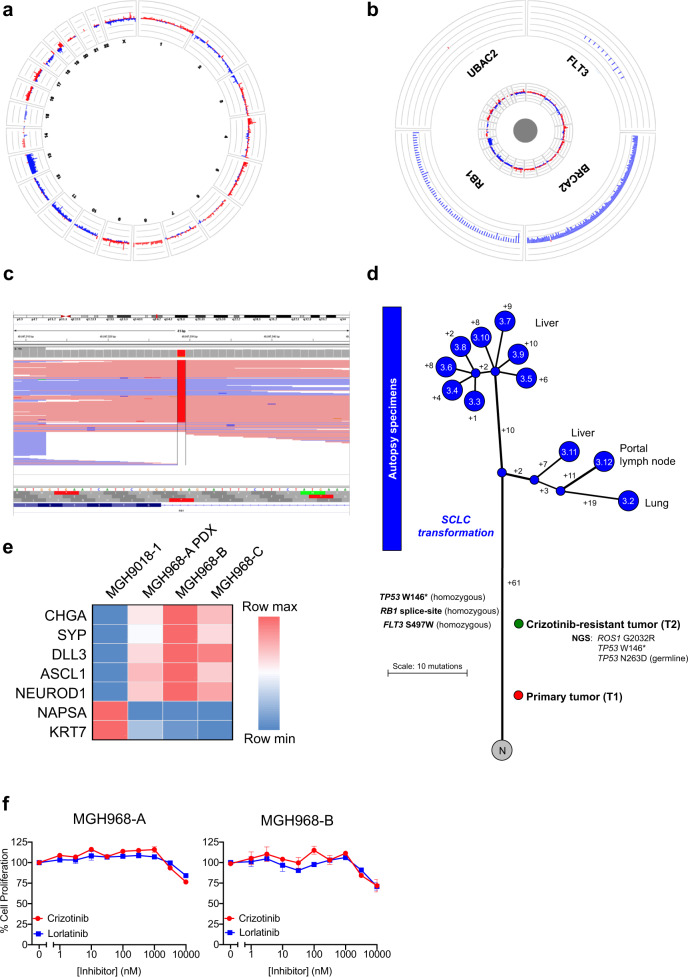
Genetic and phylogeny analysis of metastatic tumors. **a** Circos plots for a representative autopsy tumor specimen, providing a high-level overview of genomic gains (red) and losses (blue) across all evaluable probes in all chromosomes. There are diffuse losses across chromosome 13. **b** A higher magnitude view of four genes on chromosome 13 demonstrating loss of *RB1*. **c** Next-generation sequencing pile-up illustrating the presence of a splice region variant in *RB1* in the majority of the reads. **d** Branching diagram of the metastatic tumors collected at autopsy and analyzed by whole-exome sequencing. The numbers on the branches represent the number of distinct mutations (synonymous and non-synonymous). “N” refers to normal tissue. The treatment-naive tumor (T1) and the crizotinib-resistant tumor (T2) were not analyzed by whole-exome sequencing, and therefore, could not be located precisely in this diagram. **e** Decreased expression of lung epithelial genes and increased expression of neuroendocrine genes in the MGH968-A PDX, MGH968-B, and MGH968-C cell line models, as determined by quantitative RT-PCR. MGH9018-1 is a cell line derived from a crizotinib-resistant *CD74-ROS1* fusion-positive adenocarcinoma and is shown for comparison. **f** Resistance of MGH968-A and MGH968-B cells to clinically available ROS1 inhibitors. The proliferation assay was performed in triplicate, and the error bars represent the standard error of the mean.

Targeted NGS analysis of 91 cancer-related genes (SNaPshot NGS version 2; MGH, Boston, MA) in a metastatic liver sample collected at autopsy detected the two *TP53* variants (W146* and N263D) previously known from the crizotinib-resistant lung biopsy. The *TP53* W146* mutation was present at the allele fraction of 94.3% (and the N263D, likely polymorphism, was present at 95.4%), consistent with biallelic inactivation of TP53. Of note, the *ROS1* G2032R resistance mutation was no longer detected in the autopsy sample. Additional variants were detected in *RB1* (splice region/intronic variant; c.2520 3G > T) and *FLT3* (S497W; c.1490C > G); these genes had not been evaluated in the NGS analysis of the prior crizotinib-resistant lung tumor. Further analyses supported biallelic inactivation of RB1, with loss of one allele of *RB1* and the presence of the splice region/intronic variant in the remaining allele (Fig. 2a–c). By IHC, tumor cells in the original, treatment-naive biopsy demonstrated wild-type RB1 expression, but loss of TP53 expression, whereas a representative autopsy sample lacked expression of both TP53 and RB1 (Fig. 1d).

We next performed whole-exome sequencing (WES) of tumor tissue samples obtained at autopsy in order to define the genomic landscape and clonal evolution of the small cell-transformed tumor. There was insufficient tumor tissue from the initial diagnostic specimen (T1) and the crizotinib-resistant specimen (T2) for WES. A median of 37 non-synonymous mutations were identified in the autopsy tumor samples (range 33–42). Somatic variants detected in the tumor samples are shown in Supplementary Data File 1. No *ROS1* resistance mutations were detected in any of the post-mortem specimens, including *ROS1* G2032R, which had been detected in the crizotinib-resistant tumor (T2). The median total mutation burden in the autopsy tumor samples was 1.1 Mut/Mb (range 0.9–1.2 Muts/Mb), and in line with this very low overall mutational burden, there was no strong evidence for underlying mutational processes aside from a general aging signature. Clonal analysis and phylogenetic reconstruction revealed that the post-mortem metastatic sites were homogeneous, with a small number of mutations distinguishing the metastatic tumors (Fig. 2d). The median percentage of total private mutations (synonymous and non-synonymous) was 20.7% (range 15.9–27.8%). Copy number landscape was also homogeneous overall (Supplementary Fig. 1; focal amplifications and deletions shown in Supplementary Data Files 2 and 3, respectively). There was no evidence of whole-genome doubling.

### Loss of ROS1 dependency and resistance to ROS1 TKIs

The loss of expression of the *ROS1* fusion at both the mRNA and protein levels suggested a loss of dependency on ROS1 as the primary oncogenic driver as a consequence of SCLC transformation. To further examine this, we established a primary patient-derived xenograft (PDX) mouse model MGH968-A from a liver metastasis (T3.10) obtained at the time of autopsy. Histologic examination confirmed SCLC morphology with loss of RB and p53 expression (Supplementary Fig. 2A), and the *ROS1* rearrangement was confirmed by FISH (data not shown). Similar to the original tumor, solid fusion assay did not detect expression of the *SLC34A2-ROS1* transcript. In parallel, we established two cell lines (MGH968-B and MGH968-C) from two tumor samples (T3.3 and T3.6, respectively) taken from a liver metastatic lesion at autopsy. At the genomic level, we observed a fusion breakpoint within intron 31 of *ROS1* and the 3′-untranslated region of exon 13 of *SLC34A2* (Supplementary Fig. 2B, C). Consistent with the clinical tumor sample and PDX model, RB and p53 expression were lost (Supplementary Fig. 2D). We also observed decreased lung epithelial gene expression (napsin A, KRT7) and increased neuroendocrine gene expression (chromogranin, synaptophysin, DLL3, ASCL1, NEUROD1) consistent with transformation from adenocarcinoma to SCLC (Fig. 2e). We were unable to detect expression of the *SLC34A2-ROS1* fusion transcript in the established cell lines, despite the presence of the rearrangement in the genomic DNA (Supplementary Fig. 2C). However in early cultures sampled during cell line development, we detected trace expression of two alternatively spliced *SLC34A2-ROS1* fusion transcripts, the S13:R34 fusion transcript detected in the pre-treatment clinical sample as well as a S13:R32 fusion transcript (Supplementary Fig. 2B). Finally, we examined whether the SCLC-transformed MGH968 cell lines were resistant to ROS1 TKIs. Consistent with a loss of dependency on ROS1, the cell lines were insensitive to two different clinical ROS1 inhibitors (Fig. 2f). Together, these results support the notion that SCLC transformation is associated with loss of dependency on ROS1 activity and resistance to ROS TKIs.

### Low frequency of SCLC transformation in *ROS1*+ and *ALK*+ NSCLC

Given this index case of small cell transformation in *ROS1*+ NSCLC, we queried our institutional database of ROS1 TKI-resistant *ROS1*+ tumor biopsies to explore the relative prevalence of this phenomenon. A total of 65 ROS1 TKI-resistant tumor biopsies, taken from 43 unique *ROS1*+ NSCLC patients, were identified within the study time frame (Supplementary Data File 4). The majority (41/65, 63%) consisted of crizotinib-resistant biopsies, followed by lorlatinib-resistant biopsies (14/65, 22%). All but this index case retained adenocarcinoma histology. Therefore, the frequency of SCLC transformation in our ROS1 TKI-resistant cohort was low, at 2%.

*ALK*-positive (*ALK*+) NSCLC represents another established fusion-driven subset of lung cancer, for which first- and next-generation ALK inhibitors are often used sequentially in the clinic. Furthermore, ALK and ROS1 are phylogenetically related receptor tyrosine kinases. Isolated cases of small cell transformation associated with ALK TKI resistance have been reported^22–29^; however, the prevalence of SCLC transformation in *ALK*+ lung cancer has not been determined. We reviewed our institutional database of repeat tumor biopsies obtained from patients with *ALK*+ NSCLC progressing on ALK TKIs. Among 95 crizotinib-resistant tumor biopsies (derived from 91 unique patients), none were found to have small cell morphology. Of the 130 biopsy cases resistant to second-generation ALK TKIs (ceritinib: *n* = 32, from 29 patients; alectinib: *n* = 83, from 74 patients; brigatinib: *n* = 15, from 13 patients), one case had evidence of small cell transformation (0.8%). Among 38 cases resistant to the third-generation ALK TKI lorlatinib (derived from 38 unique patients), one case was documented as small cell transformed (2.6%). In total, the frequency of SCLC transformation in our cohort of any ALK TKI-resistant *ALK*+ tumors was 0.8% (2/263), and the frequency among *ALK*+ tumors resistant to next-generation ALK TKIs was 1.2% (2/168).

## Discussion

In this study, we present the analysis of metastatic tumor samples collected from a patient with advanced *ROS1*+ NSCLC who had received multiple ROS1 inhibitors during her treatment course. Evidence of small cell transformation was observed in all metastatic tumor samples harvested at autopsy. Our case indicates that histologic transformation to SCLC can occur in TKI-resistant *ROS1*+ NSCLC and highlights its importance as a relevant resistance mechanism across multiple subsets of oncogene-driven lung cancer. While small cell transformation has been reported in *EGFR*-mutant and *ALK*+ NSCLC^19–30^, it has not, to our knowledge, been reported as a mechanism of resistance in *ROS1*+ lung cancer. In patients relapsing on ROS1 inhibitors, the possibility of small cell transformation should therefore be considered; and given the limitation of liquid biopsies (i.e., circulating tumor DNA analyses) in capturing tumor histology, repeat tumor biopsies should be pursued if feasible at the time of progression on ROS1 inhibitors.

It is noteworthy that the frequency of small cell transformation in *ROS1* and *ALK* fusion-positive lung cancers appears relatively low (2% and 0.8%, respectively, in our study) compared to that observed in *EGFR*-mutant lung cancer (3–10%)^16–21^. The relative prevalence of small cell transformation in different subsets of oncogene-driven lung cancers needs to be validated in larger cohorts. Whether *EGFR*-mutant versus fusion-driven lung cancers are fundamentally different in terms of tumor cell plasticity or the differentiation state of the cell of origin, both of which may impact propensity for lineage changes, is unknown.

In addition, our analyses highlight the genetic underpinnings of the transformed *ROS1*+ lung cancer in this patient. Consistent with prior reports in *EGFR*-mutant lung cancer, small cell transformation was associated with inactivation of *TP53* and *RB1*^20^. Interestingly, while the *ROS1* fusion was retained throughout the evolutionary trajectory of the tumor (as confirmed by FISH), its expression was lost at the RNA and protein level, and the *ROS1* G2032R resistance mutation previously detected in a crizotinib-refractory tumor was not identified in the transformed tumors at autopsy. These findings suggest that the TKI-resistant SCLC and the preceding adenocarcinoma shared a common clonal origin, with early divergence of the adenocarcinoma that then went onto acquire *ROS1* G2032R (and was subsequently treated with SBRT and ablation), and of the clone that transformed into small cell and went onto seed all of the metastatic sites. The loss of ROS1 expression also parallels the loss of EGFR expression observed in small cell-transformed *EGFR*-mutant lung cancer^19^, raising the possibility that the retention of original oncogenic signaling may be incompatible with transformation to SCLC. As an alternative hypothesis, the loss of ROS1 expression could be a consequence of the diminished activity of the SLC34A2 promoter in the transformed small cell compared to adenocarcinoma (Supplementary Fig. 3).

Unexpectedly, genomic analyses of multiple post-mortem tumors revealed a remarkable degree of homogeneity in the mutational and copy number landscape across distinct metastatic sites at autopsy. These results stand in contrast to the published TRACERx study, which demonstrated high intratumor heterogeneity in early stage, predominantly smoking-related NSCLC^34,35^. The genetic homogeneity observed in this transformed *ROS1*+ case raises the possibility of a “hard” clonal sweep and subsequent diminution of genomic diversity (driven by the dominant, small cell-transformed clone). Based on the single mechanism of resistance, we speculate that the patient could have derived significant benefit from an active agent had one been available to target critical therapeutic vulnerabilities in the transformed tumor. Of note, at present, the optimal therapy for patients with transformed SCLC remains to be determined. While responses to platinum-etoposide and taxanes have been noted in small cell-transformed *EGFR*-mutant lung cancer, these responses are typically short-lived, underscoring the need for more effective treatments^21^. Further investigation will be needed to better understand the genomic and non-genomic (e.g. transcriptome and epigenome) landscape of small cell-transformed *ROS1*+ (and other oncogene-driven) lung cancer and to identify effective therapeutic opportunities.

## Methods

### Collection of samples

Patients provided written informed consent for the collection of tumor and plasma samples. Biopsies and molecular testing were performed in accordance with Institutional Review Board-approved protocols at MGH. Rapid autopsy was performed within 2 h post mortem.

### Histopathology and IHC

Hematoxylin and eosin staining was performed on 5-μm sections generated from formalin-fixed, paraffin-embedded tumor tissue. All pathology slides were reviewed by a pathologist with expertise in lung cancer pathology. Following immunostains were performed using Leica Bond III automation: ROS1 (clone D4D6, 1:200, Cell Signaling Technology, Danvers, MA), CK7 (clone OV-TL 12/30, 1:400, Cell Marque, Rocklin, CA), TTF-1 (clone 8G7G3/1, Cell Marque, Rocklin, CA), synaptophysin (27G12, Leica Biosystems, Danvers, MA), chromogranin (5H7, Leica Biosystems, Danvers, MA), TP53 (DO-7, Leica Biosystems, Danvers, MA), and RB (1F8, Bio SB, Santa Barbara, CA). All antibodies except ROS1 and CK7 were ready to use (pre-diluted by the company); ROS1 and CK7 antibodies were used at dilutions indicated above.

### Fluorescence in situ hybridization

*ROS1* FISH was performed using a break-apart approach as previously described, and scored as positive if more than 15% of tumor cells demonstrated split signals^4^.

### Targeted and WES

Targeted NGS testing on the crizotinib-resistant and autopsy tumor biopsies were pursued using the MGH NGS platform, which uses anchored multiplex polymerase chain reaction (PCR) to detect single-nucleotide variants and insertions/deletions within 39 cancer-related genes (version 1) or 91 cancer-related genes (version 2), respectively^36^. Targeted NGS to detect fusion transcripts was performed using the MGH Solid Fusion Assay platform, which employs targeted RNA sequencing with anchored multiplex PCR to detect fusion transcripts involving 12 cancer-related genes, including *ROS1*^36^.

For WES, genomic DNA was extracted from frozen tumor samples. Whole-exome capture libraries were constructed from 100 ng of extracted tumor and normal DNA. Ligated DNA was size-selected for lengths between 200 and 350 bp and subjected to exonic hybrid capture using SureSelect v2 exome bait (Agilent). Samples were multiplexed and sequenced on Illumina HiSeq flow cells (paired-end 76 bp reads) to an average on-target coverage depth ranging from 134–197× to178× for tumor and normal DNA, respectively. Massively parallel sequencing data were processed using two consecutive pipelines as previously described elsewhere^37^. A previously described Bayesian clustering procedure was employed for clonal evolution analysis^37^.

### Assessment for copy numbers

Segmentation and visualization of copy number profiles were achieved as follows: major and minor allelic counts were generated at a pre-defined set of common hg19 single-nucleotide polymorphism (SNP) loci. Count profiles were each normalized with respect to an independent diploid sample with the most similar noise profile. Copy number segmentation was done using circular binary segmentation. Purity and ploidy estimates were achieved by iteratively searching for the set of parameters that minimizes the total distance between each profile’s copy number segments and the nearest integer copy number state using MATLAB’s fminsearch function. Copy number variant (CNV) calls were generated using FACETS. SNP counts were generated with minimum mapping quality of 15, minimum base quality of 20, pseudo-spacing of 100, and minimum read count of 25. Copy number data were segmented using window size of 1000, VAF threshold of 0.3 and cval of 300. Focal CNVs were determined as copy number segments of total size <10 Mb and total copy state ≥5 or ≤1 for amplifications and deletions, respectively.

### Cell line, antibodies, and reagents

Using methods previously described^38^, the MGH9018-1R cell line was established from a post-crizotinib pleural fluid of a *CD74-ROS1* fusion-positive NSCLC patient. The MGH968-A PDX model (lab ID: MGH986-1.13) was established from a liver metastasis (T3.10) obtained at the time of autopsy by subcutaneous implantation into NSG mice (Jackson Labs), followed by two serial passages. All mouse studies were conducted through Institutional Animal Care and Use Committee-approved animal protocols in accordance with institutional guidelines. The MGH968-B (lab ID: MGH968-1.2) and MGH968-C (lab ID: MGH968-1.5) cell lines were established from liver metastases (T3.3 and T3.6) of the same autopsy case. The MGH968-B cell line was cultured in RPMI-1640 (Lonza) with 10% fetal bovine serum (FBS) plus 1% penicillin and streptomycin (Gibco) and rock inhibitor media, the MGH968-C cell line was cultured in DMEM/F12 with 10% FBS plus 1% penicillin and streptomycin, and the MGH9018-1R cell line was cultured in RPMI-1640 with 10% FBS plus 1% penicillin and streptomycin. All cell lines were maintained in humidified incubators with 5% CO_2_ at 37 °C. Mycoplasma testing was routinely performed on all the cell lines. The PDX and the cell lines were sequenced to confirm the presence of *SLC34A2-ROS1* fusion. Primers used for genomic DNA (gDNA) were: F: 5′-CTCCCCATTAGCGAATGAAA-3′ and R: 5′-ATCCAAAAGCTGGCAGAAGA-3′. Primers used for complementary DNA (cDNA) were: F: 5′-GTTCCCGTCGTCTTCATCAT-3′ and R: 5′-TCAATCTCCTCTTGGGTTGG-3′. Control human gDNA was purchased from Thermo Fisher. All drugs were purchased from Selleckchem. For cell culture studies, drugs were dissolved in dimethyl sulfoxide to a final concentration of 10 mmol/L and stored at −20 °C, unless otherwise specified. For western blotting, the following antibodies were obtained from Cell Signaling Technology and used at 1:1000 dilution: Rb (#9309), p53 (#9282), and β-actin (#4970). The patient-derived cell lines are available to the community upon reasonable request to, and communication with, the corresponding author.

### Cell proliferation assay

Three thousand cells were plated in triplicate into 96-well plates, 24 h before adding drugs. Cell proliferation was determined by CellTiter-Glo (Promega) 72 h after adding the drug. Luminescence was measured with a SpectraMax M5 Multi-Mode Microplate Reader (Molecular Devices).

### qRT-PCR

Cells were lysed and RNA was extracted using the Qiagen RNeasy Mini Kit according to the manufacturer’s protocol. cDNA was synthesized using SuperScript II First-Strand Synthesis kit (Thermo Scientific). Quantitative real-time PCR (qPCR) was performed using FastStart Universal SYBR Green Master (Roche) on a LightCycler 480 PCR platform (Roche). Gene expression was normalized to 18S reference. The following primer sequences were used: chromogranin A, F: 5′-CGAAGGGAAGGGAGAACAG-3′ and R: 5′-ACCACTGCCATCTCCTCCT-3′; synaptophysin, F: 5′-CCAATCAGATGTAGTCTGGTCAGT-3′ and R: 5′-AGGCCTTCTCCTGAGCTCTT-3′; DLL3, F: 5′-CAACTGTGAGAAGAGGGTGGA-3′ and R: 5′-CAGGTCCAGGCAGAGTCC-3′; ASCL1, F: 5′-CGGCCAACAAGAAGATGAGT-3′ and R: 5′-GCCATGGAGTTCAAGTCGTT-3′; NEUROD1, F: 5′-CGAATTTGGTGTGGCTGTA-3′ and R: 5′-TACAGCCACACCAAATTCG-3′; napsin A, F: 5′-AGGTCCCCAGCGATGTCT-3′ and R: 5′-GACTCGATGAAGAGGGATGC-3′; KRT7, F: 5′-CAGGCTGAGATCGACAACATC-3′ and R: 5′-CTTGGCACGAGCATCCTT-3′; actin, F: 5′-CTGTGCTATCCCTGTACGCCTC-3′ and R: 5′-CATGATGGAGTTGAAGGTAGTTTCGT-3′.

### Reporting summary

Further information on research design is available in the Nature Research Reporting Summary linked to this article.

## Supplementary information

Supplementary Figures

Supplementary Data File 1

Supplementary Data File 2

Supplementary Data File 3

Supplementary Data File 4

Reporting Summary

## Data Availability

The whole-exome sequencing dataset generated during the current study are not publicly available as these are patient samples with potentially identifiable germline SNPs and there is no patient consent for depositing this sequencing data in a public repository. However, the data are available from the corresponding author on reasonable request.
