# Supplementary material for: Small cell transformation of ROS1 fusion-positive lung cancer resistant to ROS1 inhibition
Source: NPJ Precis Oncol. 2020 Aug 3;4:21. doi: 10.1038/s41698-020-0127-9 (PMC7400592; doi:10.1038/s41698-020-0127-9)
Supplement: Supplementary file 1 — Supplementary Figures [file 41698_2020_127_MOESM1_ESM.pdf]

Supplementary Figure 1

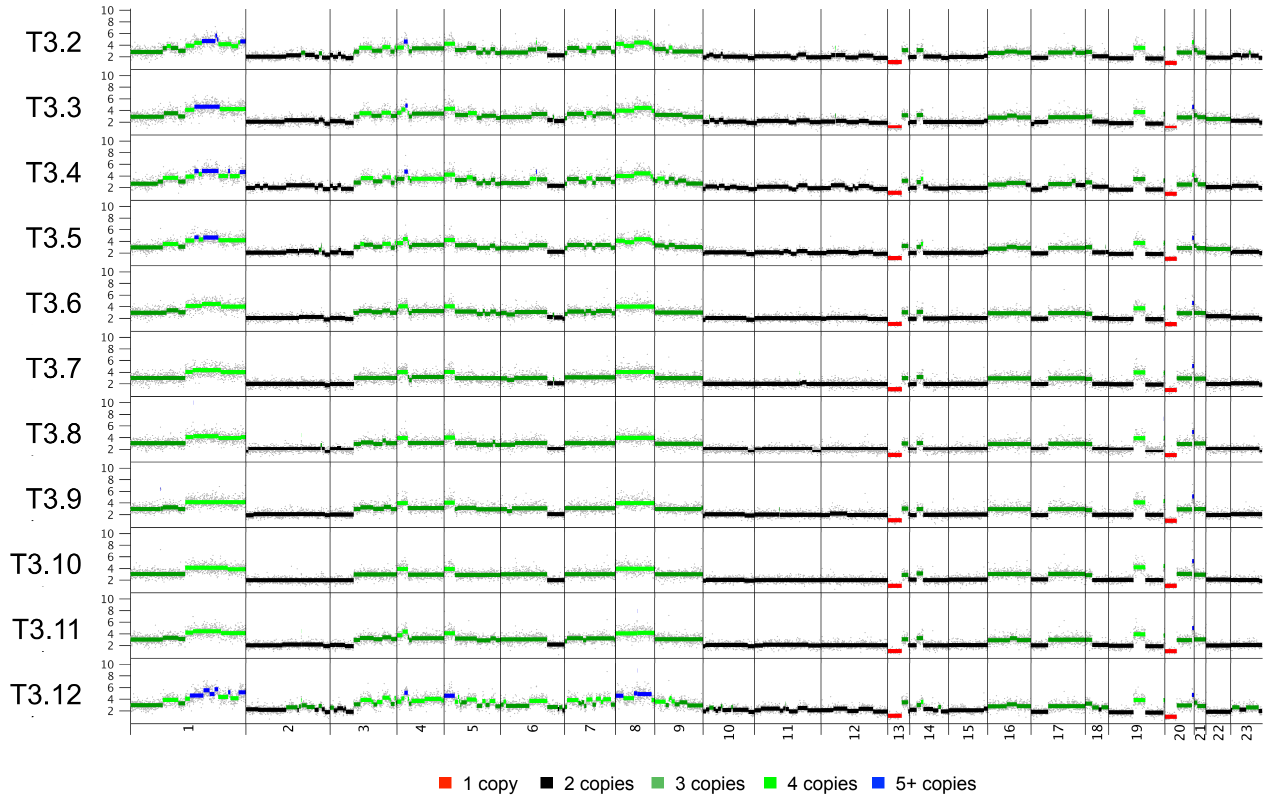

Supplementary Figure 1. Copy number landscape across the metastatic autopsy samples. Copy number profiles computed using SNP-based segmentation demonstrate low levels of heterogeneity across the autopsy specimens. Vertical lines segregate each chromosome, and different colors correspond to distinct copy numbers.

Supplementary Figure 2

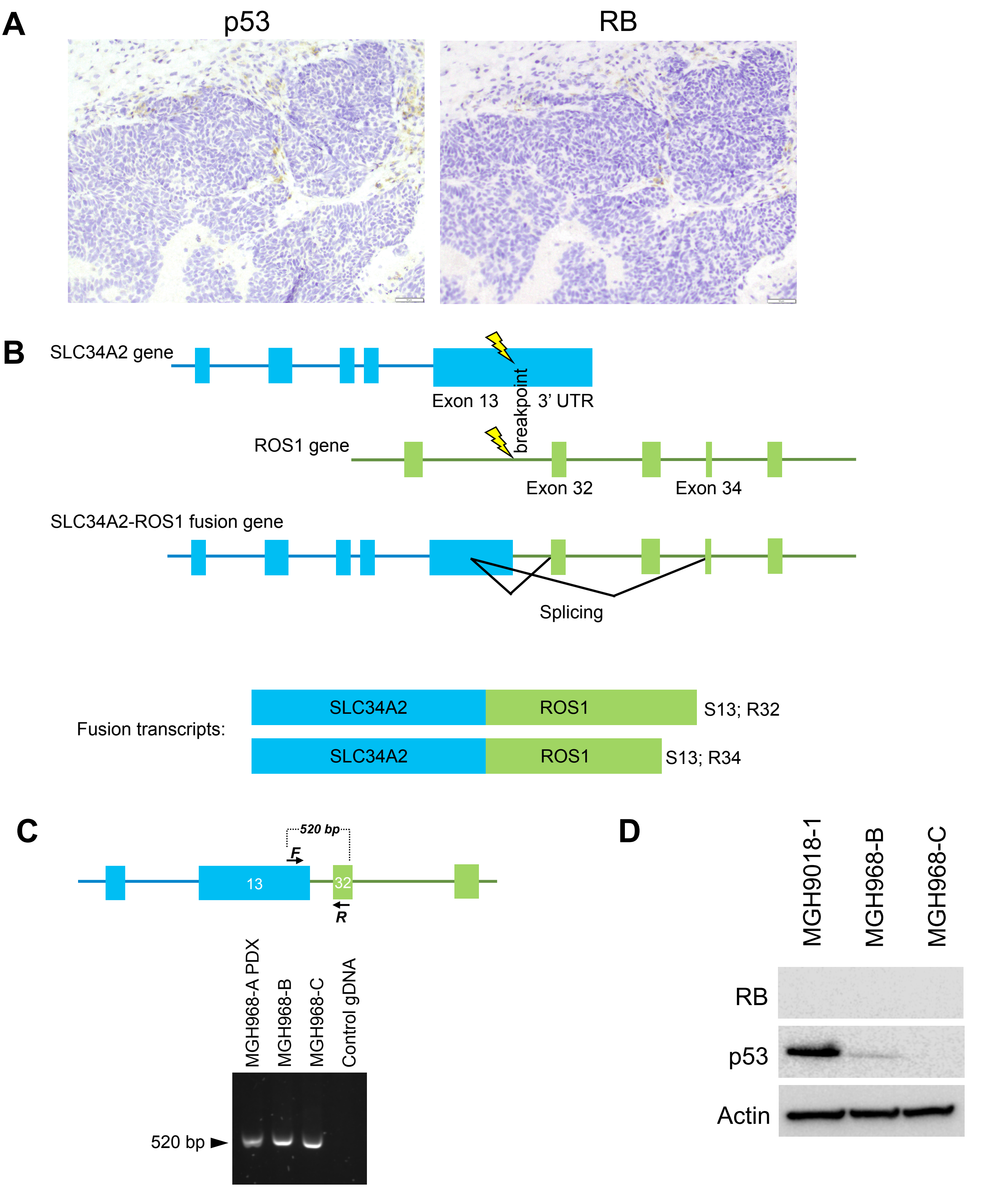

Supplementary Figure 2. Characterization of patient-derived experimental models. (A) A primary patient-derived xenograft (PDX) mouse model MGH968-A generated from an autopsy sample (T3.10) with small cell morphology and loss of TP53 and RB1 expression. (B) Schematic representing the *SLC34A2-ROS1* gene rearrangement and resulting mRNA fusion transcripts. (C) PCR assay confirms *SLC34A2-ROS1* fusion in the genomic DNA. (D) Loss of RB1 and TP53 expression in MGH968-B and MGH968-C cell lines.

Supplementary Figure 3

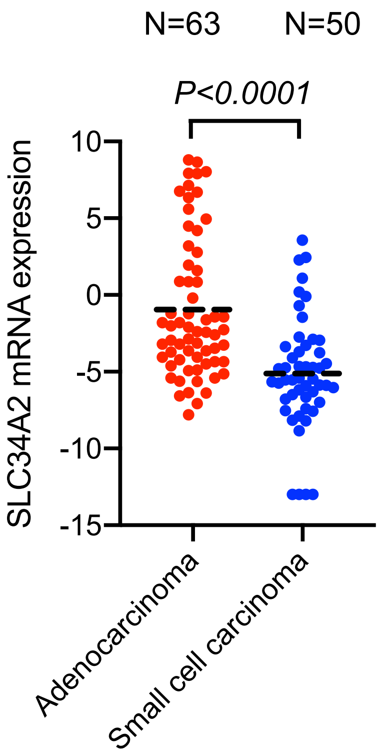

Supplementary Figure 3. Comparison of mRNA expression of SLC34A2 in adenocarcinoma (red, n=63) and small cell carcinoma (blue, n=50) lung cancer cell lines. The RNAseq dataset of cancer cell line encyclopedia (CCLE) was obtained from the CCLE portal (https://portals.broadinstitute.org/ccle). The dotted lines indicate the means and two-sided t-test was performed between two groups.
